# The impact of COVID-19-induced lockdowns on Antiretroviral-Therapy (ART) adherence by HIV/AIDs patients on ART in the city of Bulawayo in Zimbabwe

**DOI:** 10.4314/ahs.v24i2.4

**Published:** 2024-06

**Authors:** Mjabuli Jamela, Macide Artac Özdal

**Affiliations:** 1 European University of Lefke, Lefke, Northern Cyprus TR-10 Mersin, Turkey, 99010

**Keywords:** Impact of COVID-19-induced lockdowns, HIV/AIDs patients, Bulawayo, Zimbabwe

## Abstract

**Background:**

Zimbabwe has one of the highest HIV prevalence rates in the world. HIV treatment was initiated in 2004 and expanded to 94% coverage rate by the 2020.

**Objectives:**

i) to determine the level of treatment adherence during COVID-19-induced lockdowns and ii) to investigate the key determinants of adherence to ART during COVID-19-induced lockdowns.

**Methods:**

The cross sectional study involved 392 people living with HIV (PLHIV) and was conducted at nine health facilities in Bulawayo City. Data was analysed using the Shapiro-wilk test for normality, Chi-squared test, Kaiser-Meyer-Olkin (KMO), Bartlett's test, exploratory factor analysis, reliability analysis, scree plot, correlation analysis and multiple linear regression analysis.

**Results:**

94.6% of the respondents took their ARTs on time, and 90.6% did not miss any treatment review. The factors influencing treatment adherence were health systems (beta value 0.334), Family support (beta value 0.138) and knowledge/understanding of treatment (beta value 0.109). Health outcome concerns (beta value -0.194) and food security and livelihoods (beta value 0.191).

**Conclusion:**

Three factors had a positive impact on treatment adherence namely, functional health systems, family support, and knowledge or understanding of health treatment, while two factors namely health outcome concerns and food security and livelihoods negatively impacted treatment adherence.

## Introduction

The Human Immunodeficiency Virus (HIV) pandemic continues to be of global significance. Cumulative HIV-related deaths are estimated at 40.1 million^1^. In 2019, HIV infection caused 47.63 million disability-adjusted life years (DALYs) worldwide^2^. Africa is the epicentre of HIV and is home to two-thirds of the world's population of People living with HIV (PLHIV), although only 11% of the world's population lives on the continent^1^,^3^. Zimbabwe has one of the highest HIV prevalence rates in the world 1. The national HIV prevalence is estimated at 11.9% (10.7-14), the incidence rate is 0.3% (0.19-0.39), and the country has an estimated PLHIV population of 1,280,000. Risky behaviours and HIV prevalence are higher in the south-western provinces, which include the City of Bulawayo. The city has an estimated HIV prevalence rate of 13.8%, which is higher than the national average^4^,^5^.

The HIV treatment programme in Zimbabwe was launched in 2004 and has grown continuously. In 2020, 94% of PLHIV over the age of 15 were on antiretroviral therapy (ART)^4^,^6^,^7^. Due to the success in HIV treatment, Zimbabwe has increased its targets from the 90-90-90 strategy to the 95-95-95 strategy in line with the Sustainable Development Goals to be achieved by 2030^8^,^9^. The 95-95-95 strategy ensures that 95% of all PLHIV know their HIV status, 95% of PLHIV receive sustained ART and 95% of persons receiving ART will achieve viral suppression by 2025^10^. The self-reporting instrument consisted of a series of six questions that assessed adherence by evaluating forgetfulness, routine, adverse effects, and quantification of omissions. The research SMAQ adopted the interpretation of the SMAQ tool, which categorises any respondent who gave a non-adherent response as non-adherent 39.

In addition to the demographic information and treatment adherence questions from the SMAQ tool, the research questionnaire contained a total of thirty-one statements designed to determine the relationship between treatment adherence (the outcome of interest) and the factors influencing that outcome. The answers to these questions were on a five-point Likert scale (Strongly Agree, Agree, Neither Disagree nor Agree, Disagree and Strongly Disagree).

The thirty-one statements on treatment adherence were analysed using a factor analysis with Varimax rotation. Using both the Eigenvalue criterion and the scree plot, seven factors were extracted from the factor solution, as illustrated in Table 2. Factor 1 Treatment adherence (TA) (questions C27, C28, C29, C30), Factor 2 Health Education (HE) (questions C19, C20, C21, C22), Factor 3 Health Systems (HS) (Questions C23, C24, C25, C26), Factor 4 Health Outcome Concerns (HOC) (questions C3, C4, C6), Factor 5 Food Security and Livelihoods (FSL) (questions C7, C10, C11), Factor 6 Family Support (FS) (questions C12, C14), Factor 7 Knowledge/Understanding of Treatment (KT) (questions C1, C2, C5). In addition, a total of seven questions were excluded from the factor solution because they did not measure the factor under which they were summarised. As illustrated in Table 2, all factors had a Cronbach's alpha greater than 0.7, and the research concluded that all seven factors had good internal consistency 43. The statements for each factor are shown in Table 3.

### Data collection and management

The questionnaire was administered by ten trained research assistants (enumerators). The research assistants were led by a supervisor who was also responsible for ensuring data quality. The data was collected, transferred and stored by the research assistants via an online platform (KOBO Toolbox).

### Data analysis

The data was first presented in a Microsoft Excel spreadsheet in order to clean the data and perform basic analyses (development of pie charts and bar graphs etc.). The data was then imported and analysed using IBM Statistical Package for Social Science (SPSS) version 28. Custom tables were used to summarise the data for ease of interpretation and analysis. Data were tested for normality using the Shapiro-Wilk test and the Chi-squared test was used to compare variation between categorical variables. Exploratory factor analysis was used as a technique for dimension reduction. The Kaiser-Meyer-Olkin (KMO) test and Bartlett's tests were used to determine validity and to ensure that the data was suitable for Exploratory factor analysis. Correlation analysis was used to measure the relationship between the dependent variables. Statistical significance of the model was evaluated using analysis of variance (ANOVA). Multiple linear regression was used to assess the linear relationship between the explanatory variables and the response variable. The explanatory variables were: i) health systems, ii) family support, iii) knowledge/understanding of treatment, iv) health outcome concerns v) food security and livelihoods. The response variable was treatment adherence.

### Ethical considerations

Ethical approval for the research was granted by the Ethics Committee of the European University of Lefke (EUL) on 3 April 2023 (approval number BAYEK 022.01). Additional authorisation to conduct the study was granted by the Bulawayo City Council and verbal consent was obtained from PLHIV before they could participate in the study. Verbal consent was chosen as written consent was not feasible in the interest of confidentiality - this was raised as a concern during pretesting by potential respondents. Consenting adult PLHIV were interviewed, while consenting legal guardians of minors under the age of 18 were interviewed in their place. The decision not to interview minors directly was made during the pretesting. The legal guardians did not agree to the minors being interviewed. The legal guardians were therefore interviewed, as they are responsible for ensuring that the minors take their medication. The research assistants were trained in research ethics, signed non-disclosure (confidentiality) agreements and did not collect personally identifiable information.

## Results

### Demographic characteristics of participants

A total of 392 PLHIV from the catchment areas of nine Bulawayo City Council clinics were interviewed as part of the study. 297 (75.8%) were adult PLHIV, while 95 (24.2%) of the respondents were carers or guardians of minors living with HIV. More than half 234 (59.7%) of the respondents were females and 158 (40.3%) were males. The age of the respondents ranged from 6 to 82 years. The average age was 39.4 years, the median was 39 years and the standard deviation was 14 years. 214 (54.6%) of the respondents had completed secondary school, 83 (21.2%) had completed tertiary education, (65) 16.6% had completed primary school, and 30 (7.7%) had no formal education. 187 (47.7%) of the respondents were married, 134 (34.2%) were single, 46 (11.7%) were widowed and 24 (6.4%) were divorced. 367 (93.6%) of respondents lived in high-density suburbs, (13) 3.3% lived in rural areas (peri-urban) 7 (1.8%) and 5 (1.3%) reported living in medium-density and low-density suburbs. The average household size of the study participants was 4 people and the standard deviation of household size was 1.8.

### Treatment adherence during the lockdown period

94.6% of respondents said they took their Antiretroviral drugs (ARVs) on time, while 5.4% said they did not take their ARVs on time during the COVID-19 lockdown period. Cross-tabulation of the data showed that 4.3% of females and 7% of males did not take their ARVs on time. A chi-square test of independence was performed to examine the association between respondent gender and forgetting to take ARVs on time. The study established that there was no significant association between these variables, X2 (1, N = 392) = 1.345, p = 0.246.

90.6% of respondents stated that they had not missed a treatment review appointment, while 9.4% emphasised that they had missed at least one treatment review during the COVID-19 lockdown period. 7.3% of females missed treatment reviews, while 12.7% of males missed treatment reviews. 96.2% of respondents said that they did not stop taking ARVs when they felt bad or sick, while 3.8% indicated that they stopped taking ARVs when they felt sick. 3.8% of PLHIV highlighted that sometimes they forgot to take their ARVs. 96.2% took their ARVs as prescribed (correct amount and number of times). The study also established no significant relationship between the respondents' gender and forgetting to take ARVs, X2 (1, N = 392) = 0.322, p = 0.571.96.4% of study participants reported that no entire day went by without taking their ARVs during the study period. The study did not collect data on the pre-COVID-19 period of PLHIVs. However, the study analysed data from the Ministry of Health and Child Care data which exhibited a decrease in PMTCT coverage from 94% to 87% in 2019 and 2020 respectively. The same data revealed an increase in ART coverage from 91% to 94% over the same period^5^.

### Perceptions on the key factor impacting treatment adherence

The Kaiser-Meyer-Olkin Measure of Sampling Adequacy (KMO) and the Bartlett's Test of Sphericity were used to test for validity. The KMO measure of sampling adequacy was 0.798 and the P-value for the Bartlett's test of sphericity was <0.001. As the data were considered valid, the researchers conducted an exploratory factor analysis. Exploratory factor analysis was used as a dimension-reduction technique, as shown in Table 1^42^.

**Table 1:** Factor loadings for the study variables

		Factor loadings						
Code	Variable name	TA	HE	HS	HOC	FSL	FS	KT
C28	There were times when I did not take the correct amount of ARVs advised by health workers.	0.859	-0.096	-0.109	0.098	-0.058	-0.133	-0.043
C27	There were times when I did not have adequate access to ARV	0.846	-0.088	-0.092	0.096	-0.056	-0.019	-0.073
C29	There were times that I did not take ARVs at the correct times	0.791	-0.105	-0.201	0.085	0.314	-0.091	-0.02
C30	There were times that I did not take ARVs for a day or more	0.787	-0.044	-0.151	0.182	0.333	-0.028	-0.104
C31	I defaulted from the ARVs (Anti-Retroviral Therapy)	0.618	-0.151	-0.194	-0.302	-0.13	-0.173	-0.104

C20	Health workers continued providing individualized health education and counselling	-0.016	0.857	0.116	0.106	0.086	0.057	0.017
C21	Health workers continued communicating and treated me politely with me	-0.125	0.778	0.188	0.069	-0.051	0.031	0.11
C22	The health education messages provided by health workers were clear and consistent.	-0.096	0.755	0.12	0.013	0.025	0.058	0.202
C19	Health workers continued providing appropriate group health education messages.	-0.148	0.73	0.266	0.162	-0.029	0.063	0.088

C23	The HIV treatment facility/Health center was open during normal working hours.	-0.095	0.078	0.762	-0.071	0.054	0.08	0.058
C26	HIV treatment/Health centres continued to offer free HIV treatment services (Service including HIV testing and counselling, initiation, provision of ART etc.)	-0.158	0.233	0.715	0.089	-0.138	-0.034	0.203
C25	Medication (ARVs and medication for Opportunistic infections) were in stock	-0.238	0.206	0.709	0.256	-0.131	-0.046	0.012
C24	Health workers were available at the HIV treatment/Health Center	-0.152	0.281	0.692	0.107	0.013	0.086	-0.022

C3	I was concerned about HIV complications	0.049	0.105	0.267	0.856	0.038	-0.093	0.006
C4	I was concerned about COVID-19 complications	0.089	0.145	0.157	0.803	0.075	-0.061	0.101
C6	I spent a lot of time thinking about the side effects of HIV treatment	0.123	0.069	-0.199	0.696	0.255	0.176	-0.079

C7	My household income significantly increased.	0.171	-0.08	-0.124	0.135	0.819	0.136	0.023
C11	My household had access to adequate nutrition which is necessary for my treatment.	0.169	0.055	-0.23	0.095	0.791	0.031	0.149
C10	My household received financial support (e.g. from the government, NGOs, relatives etc.)	-0.169	0.078	0.331	0.086	0.675	0.087	-0.017

C14	My family members monitored and supported my treatment/taking of ARVs	-0.149	0.085	0.044	-0.004	0.05	0.936	0.047
C12	My HIV treatment was a top priority to my family	-0.143	0.092	0.053	-0.006	0.165	0.921	0.006

C2	I understood how ARVs work to suppress HIV	-0.076	0.147	0.026	0.081	0.135	0.005	0.785
C1	I had enough knowledge of HIV and COVID-19	-0.179	0.177	0.134	0.206	0.041	-0.083	0.74
C5	I believed that HIV treatment (ART) is an effective safe treatment	0.001	0.045	0.035	-0.192	-0.042	0.108	0.687

**Table 2:** Unique variance and internal consistency. Original Table

Statistical test	TA	HE	HS	HOC	FSL	FS	KT
Eigenvalues	5.708	3.440	2.352	1.720	1.520	1.211	1.084
Percentage of variance explained	23.785	14.335	9.801	7.165	6.334	5.045	4.517
Cronbach's Alpha Coefficient	0.789	0.837	0.789	0.765	0.708	0.915	0.765

TA: Treatment adherence, HE: Health Education, HS: Health systems, HOC: Health Outcome concerns, FSL: Food security and livelihoods, FS: Family support, KT: Knowledge/understanding of treatment.

**Figure 1:**
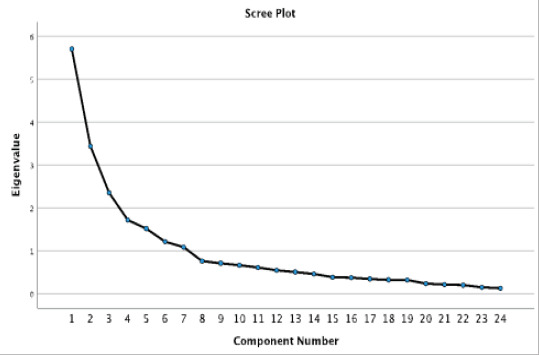
Scree plot

A custom table (Table 3) was used to summarise the descriptive data of the seven factors that emerged from the factor analysis with Varimax rotation and the Scree plot (Fig 1). In addition to summarising the data, the use of custom tables enabled the researcher to present the analysis in the form of, production-ready tables with presentation quality and analytical potential. In addition, the two lower scales (Strongly disagree and Disagree) scales were combined for easier interpretation and data presentation, and the upper scales (Agree and Strongly disagree) were combined for the same reason^38^.

**Table 3:** PLHIVs' Perceptions on factors influencing treatment adherence. Original table

Factor/Item		Question	Strongly Disagree/Disagree	Neither Disagree nor Agree	Agree/Strongly agree	Mean	SD
1. Treatment adherence (TA)	C27	There were times when I did not have adequate access to ARV	350 (89%)	7(1.8%)	35(9%)	1.86	0.879
	C28	There were times when I did not take the correct amount of ARVs advised by health workers.	348(89%)	6(2%)	38(10%)	1.86	0.89
	C29	There were times that I did not take ARVs at the correct times	246 (63%)	20 (5%)	126 (32%)	2.34	1.284
	C30	There were times that I did not take ARVs for a day or more	270(67%)	25(6%)	97(25%)	2.19	1.213
	C31	I defaulted from the ARVs (Anti-Retroviral Therapy)	377(96%)	5(1%)	10(3%)	1.41	0.661

2. Health Education (HE)>	C19	Health workers continued providing appropriate group health education messages.	11(3%)	44(11%)	337(86%)	4.17	0.764
	C20	Health workers continued providing individualized health education and counselling	14(4%)	51(13%)	327(83%)	4	0.743
	C21	Health workers continued communicating and treated me politely with me	13(3%)	46(12%)	333(85%)	4.08	0.767
	C22	The health education messages provided by health workers were clear and consistent.	15(4%)	40(10%)	337(86%)	4.07	0.737

3. Health Systems (HS)	C23	The HIV treatment facility/Health center was open during normal working hours.	6(1.6)	15(4%)	371(95%)	4.2	0.612
	C24	Health workers were available at the HIV treatment/Health Center.	9(2%)	26(7%)	357(91%)	4.16	0.658
	C25	Medication (ARVs and medication for Opportunistic infections) were in stock	13(3%)	99(25%)	280(71%)	4.02	0.871
	C26	HIV treatment/Health centres continued to offer free HIV treatment services	3(1%)	20(5%)	369(94%)	4.3	0.61

4. Health Outcome Concerns (HOC)	C3	I was concerned about HIV complications	108(28%)	30(8%)	254(65%)	3.57	1.218
	C4	I was concerned about COVID-19 complications	95(24%)	49(13%)	248(63%)	3.54	1.135
	C6	I spent a lot of time thinking about the side effects of HIV treatment	163(42%)	50(13%)	179(45%)	3.07	1.215

5. Food Security and Livelihoods (FSL)	C7	My household income significantly increased.	257(66%)	57(15%)	78(20%)	2.06	1.281
	C10	My household had access to adequate nutrition which is necessary for my treatment.	87(22%)	76(19%)	229(58%)	3.35	1.175
	C11	My household received financial support.	245(63%)	17(4%)	130(33%)	2.26	1.423

6. Family Support	C12	My family members monitored and supported my treatment/taking of ARVs	37(10%)	23(6%)	332(85%)	4.02	0.903
	C14	My HIV treatment was a top priority to my family	34(9%)	25(6%)	333(85%)	4.03	0.896

7. Knowledge/Understanding of the Treatment (KT)	C1	I had enough knowledge of HIV and COVID-19	10(3%)	28(7%)	354(90%)	4.21	0.68
	C2	I understood how ARVs work to suppress HIV	5(1%)	20(5%)	367(94%)	4.23	0.61
	C5	I believed that HIV treatment (ART) is an effective safe treatment	1(0%)	24(6%)	367(94%)	4.43	0.6

SD: Standard deviation

The Pearson correlation was used to understand the relationship between each of the independent variables and the dependent variables. The relationship between treatment adherence (TA) and health education (HE) was tested using the Pearson product-moment correlation coefficient yielded r = 0.264, P value = 0.000 N = 392. The Person product-moment correlation test results for the relationship between treatment adherence (TA) and Health systems (HS) yielded r = 405, P value = 0.000, and N = 392. A positive correlation between treatment adherence (TA) and health outcomes Concerns (HOC) concerns was realised r = -0.151, P value = 0.003, N= 392. The relationship between treatment adherence (TA) and food security and livelihoods (FSL) was tested using the Person product-moment correlation. A positive correlation was found between treatment adherence (TA) food security and livelihoods (FSL) the two variables r = -0.212, P value = <0.000, N = 392. The relationship between treatment adherence (TA) and family support (FS) was tested using the Pearson product-moment correlation coefficient. The test yielded r = 0.239, P value = <0.000 N = 392. The relationship between treatment adherence (TA) and Knowledge/understanding of the treatment (KT) was tested using the Pearson product-moment correlation coefficient. The test yielded r = 0.206, P value = <0.000 N = 392. All P values were less than 0.05 which meant that all the individual independent factors had a statistically significant relationship with the dependent variable treatment adherence (TA).

The regression model was tested for statistical significance using ANOVA. The model was found to be statistically significant, as evidenced by the P value = 0.000, which was below the threshold value of 0.05^45^-^47^. The regression model was tested and met all assumptions of regression, namely linearity, homoscedasticity, independence and normality.

Multiple linear regression is sensitive to outliers, because it is a mathematical maximisation model and outliers distort the results. In line with Tanbachnick and Fidell's (2001) recommendation to delete outliers if they are less than 5%, the researcher deleted fourteen outliers 48. After excluding the outliers, the maximum Mahalanobis distance for the model was 20.597, which is below the critical value of 22.460, making the model suitable for regression analysis.

In the regression model (Table 4), five independent factors were found to be significant predictors of the dependent variable treatment adherence (TA). These were Health Systems (HS), Family Support (FS), Knowledge/understanding of treatment (KT), Health outcome concerns (HOC), and food security and livelihoods (FSL).

**Table 4:** Regression Analysis. Original table

	Unstandardized Coefficients		Standardized Coefficients			95.0% Confidence Interval for B		Collinearity Statistics	
Model	B	Std. Error	Beta	t	Sig.	Lower Bound	Upper Bound	Tolerance	VIF
1 (Constant) Health Education and Communication	0.638 0.120	0.448 0.082	0.081	1.424 1.469	0.155 0.143	-0.243 -0.041	1.520 0.281	0.620	1.613
Health Systems	0.544	0.088	0.344	6.200	0.000	0.371	0.716	0.618	1.617
Health Outcome Concerns	-0.162	0.042	-0.194	-3.867	0.000	-0.244	-0.079	0.761	1.315
Food Security and Livelihoods	-0.150	0.039	-0.191	-3.851	0.000	-0.227	-0.073	0.774	1.292
Family Support	0.176	0.044	0.183	4.040	0.000	0.091	0.262	0.929	1.077
Knowledge of the Treatment	0.206	0.086	0.109	2.397	0.017	0.037	0.375	0.917	1.090

a. Dependent Variable: Treatment Adherence

## Discussion

The study found that ART adherence remained high during the COVID-19-induced lockdown. The perceptions of PLHIV on factors influencing ART adherence are summarised in Table 3. The study further analysed the data on perceptions of PLHIV and identified five factors that contributed to treatment adherence, namely Health Systems (HS), Family Support (FS), Knowledge/understanding of treatment (KT), Health outcome concerns (HOC), and food security and livelihoods (FSL). Of these factors, two factors Health outcome concerns (HOC) and food security and livelihoods (FSL), were negatively associated with treatment adherence during the COVID-19-induced lockdown.

PLHIV should receive continuous medication to achieve optimal clinical benefit^50^. The minimum threshold for adherence to ART treatment has traditionally been set at 95%; however, new studies have a lower threshold of ≥90%^28^-^30^. This lower threshold is largely attributed to the improved pharmacokinetic profiles of modern ARVs 51. The results of this study revealed that despite the challenges posed by COVID-19-induced lockdown measures, ART adherence remained high, with more than 90% of respondents taking their ARVs on time, did not misstheir treatment reviews, did not forget taking their ARVs and took their ARVs as prescribed. The study data indicates that 0.5% of respondents defaulted due to COVID-19-induced lockdown factors. HIV estimates for the country show a decline in PMTCT coverage from 94% in 2019 (pre-COVID-19) to 87% in 2020 during the first year of the COVID-19 pandemic and an increase in HIV-related mortality when comparing 2019 and 2020 HIV levels. The Ministry of Health and Child Care attributed this to COVID-19-related disruptions in health services. In terms of ART coverage, the same report shows an increase in ART coverage from 91% in 2019 to 94% in 2020 in the first year of the COVID-19 pandemic. This can be explained by the robust ART programme in the country 5.

Although the treatment threshold of ≥90% was met, one study recommended that healthcare professionals should continue to promote treatment adherence^28^. In June 2022, the National AIDS Council (NAC) announced at a press conference that Zimbabwe had reached its 95-95-95 targets. In Zimbabwe, 96% of people living with PLHIV know their status, 97% of these are on treatment, and of those who are on treatment, 95% are virally suppressed^49^. The announcement aligns with this study, which reveals that despite COVID-19-related disruptions to health services, treatment adherence levels are high.

Health systems faced COVID-19-related disruptions that increased HIV-related mortality from 20,100 in 2019 to 22,200 in 2020 and reduced access to PMTCT from 94% to 87% coverage^5^. Despite these disruptions, the study found that Health Systems (HS) remained the strongest driver or predictor of treatment adherence (TA) based on the Pearson product-moment correlationtest results. Health systems continued to provide ART services, ARVs were in stock most of the time, and clinics were open which contributed to adherence. Innovations in ART service delivery, such as telemedicine, have aided sustenance access to ART^52^. Stress increased among PLHIVs, there was disruption in the provision of health services and an apparent breach of confidentiality in the provision of ARVs as ART clinics were done in the open due to COVID-19 prevention protocols which hindered access to ART^53^.

PLHIV need a healthy and supportive environment 48. The second strongest factor for treatment adherence (TA) was Family Support (FS). Family members considered HIV treatment a top priority and monitored and supported PLHIV's adherence to ART. This finding confirms some studies that revealed that family support improes treatment adherence and treatment outcomes in chronic diseases^56^,^57^,^58^. Adherence was higher in patients whose family members monitored medication and provided spiritual support 59.

Knowledge/understanding of treatment (KT) was identified as the least strong positive statistically significant factor contributing to treatment adherence (TA) during the COVID-19 lockdown period. Other studies have found that knowledge is a predictor of adherence and that providing more information to patients (and their families) improves treatment adherence 60,61. Another study concluded that patients with more knowledge were twice as likely to adhere to ART^63^.

Several studies reveal the relationship between Food security and livelihoods (FSL) and treatment adherence (TA). Food insecurity is causally related to non-adherence to treatment. Reasons include worsening hunger and side effects when ART is taken in the absence of food, and ART competes for resources 63. Individuals with low to very low food security were significantly non-adherent compared to those with higher food security 65. ZIMSTAT (2021) and this study concur that COVID-19-induced lockdowns lowered household incomes and food security^64^.

Health outcome concerns (HOC) were found to be negatively related to treatment adherence (TA) as PLHIV were worried, anxious and depressed. This could be related to limited access to information on COVID-19 as well as myths and misconceptions (including COVID-19 and HIV co-infection) that affected treatment adherence. Other studies came to similar conclusions: Anxiety and depression increased the likelihood of non-adherence to antihypertensive medication^66^. People suffering from depression are three times more likely to be non-compliant with medical treatment recommendations^67^-^69^. A meta-analysis of 31 studies and 18,245 participants concluded that the likelihood of not adhering to treatment recommendations was 1.79 times higher than in non-depressant patients^70^.

## Limitations

The locations of the study were selected through convenience sampling, which reduced the representativeness and generalisability of the study. The study was conducted postfactum and was therefore based on the memory of events that occurred over a long period of time. This may have led to bias, as respondents may not have been able to recall some of their experiences accurately. The study was conducted on the basis of self-reporting, as access to clinical records was not possible, which may have introduced bias. The study also used the SMAQ tool, which has been recommended by other researchers to measure treatment adherence. To mitigate self-reporting bias, the study triangulated the information with extant literature.

## Conclusion

The level of treatment adherence in the study area remained significantly high despite the decline caused by the COVID-19-lockdown-induced disruption of services 5. Most PLHIV continued to access ART services despite access being restricted by the COVID-19 lockdown. The study also additionally, identified three positive factors for treatment adherence, namely Health Systems (HS), Family Support (FS), Knowledge/understanding of treatment (KT), and two factors that negatively affected treatment adherence (TA) namely Health outcome concerns (HOC) and food security and livelihoods (FSL). These five factors influenced adherence to HIV treatment during the COVID-19-induced lockdown period. The findings of this study can be used by health workers, academia, public health professionals and policymakers to strengthen the design, implementation, and monitoring of the ART programme in Zimbabwe. In addition, the findings can be used to improve emergency preparedness for future public health emergencies and lockdown policy planning to mitigate their impact on the management of chronic disease programmes.

Governments should invest more resources towards strengthening their health systems, as this will make the health system more resilient to shocks such as the COVID-19 pandemic. Governments should also invest in safeguarding the food security and livelihoods of people with chronic illnesses as they are relatively more vulnerable. ART programmes should also aim to promote family support and ensure that ART patients and their caregivers have sufficient knowledge and understanding of treatment. Due to the vulnerability of PLHIV to mental health conditions such as anxiety, ART programmes should integrate mental health and psychosocial support (MHPSS). ART programmes should not stand alone, but should refer pathways to other complementary services such as MHPSS, income-generating projects, life skills training and gender programmes. Future research could focus on understanding the COVID-19-related psychosocial factors that have impacted people with PLHIV, as well as evaluating the COVID-19 policy formulation process.

## References

[1] World Health Organisation (2021). HIV/AIDS factsheet.

[2] Liu W, Yang C, Chen Z, Lei F, Qin J J, Liu H, Ji Y X, Zhang P, Cai J, Liu Y M, She Z G, Zhang X J, Li H (2019). Global death burden and attributable risk factors of peripheral artery disease by age, sex, SDI regions, and countries from 1990 to 2030: Results from the Global Burden of Disease study. Atherosclerosis.

[3] Goliber T (2002). The status of the HIV AIDS pandemic in Sub Sahara Africa.

[4] Ministry of Health and Child Care, National AIDS Council Global AIDS Response Programme Report 2020-UNAIDS, GAM Zimbabwe Country Report, MoHCC and NAC.

[5] Ministry of Health and Child Care (2021). Zimbabwe National and Sub-National HIV Estimates Report.

[6] Zimbabwe National Statistics Agency and ICF International (2016). Zimbabwe Demographic and Health Survey 2015. Final Report.

[7] UNAIDS (2020). UNAIDS Data 2020.

[8] Hakim Avi J (2021). Progress Toward the 90-90-90 HIV Targets in Zimbabwe and Identifying Those Left Behind. JAIDS Journal of Acquired Immune Deficiency Syndromes.

[9] Avert Organisaiton (2022). Understanding HIV epidemic: At a glance HIV in Zimbabwe.

[10] Frescura L, Godfrey-Faussett P, Feizzadeh A A (2022). Achieving the 95 95 95 targets for all: A pathway to ending AIDS. PLoS One.

[11] Ministry of Health and Child Care (2020). Zimbabwe National HIV Strategic Plan. 2021-2025.

[12] Haileamlak A (2021). The impact of COVID-19 on health and health systems. Ethiop J Health Sci.

[13] World Bank (2022). World Development Report 2022: Chapter 1. The economic impacts of the COVID-19 crisis.

[14] Centre for disease control (2021). Basics of COVID-19.

[15] Menendez C, Gonzalez R, Donnay F, Leke RGF (2020). Avoiding indirect effects of COVID-19 on maternal and child health. Lancet Glob Health.

[16] Passos L, Prazeres F (2020). Impact on mental health due to COVID-19 pandemic: cross-sectional study in Portugal and Brazil. Int J Environ Res Public Health.

[17] Zhamantayev O, Kayupova G, Nukeshtayeva K, Yerdessov N, Bolatova Z, Turmukhambetova A (2023). COVID-19 Pandemic Impact on the Maternal Mortality in Kazakhstan and Comparison with the Countries in Central Asia. International Journal of Environmental Research and Public Health.

[18] World Health Organisation (WHO) (2021). Attacks on health care in the context of COVID-19.

[19] Mbunge E (2020). Effects of COVID-19 in South African health system and society: An explanatory study. Diabetes & Metabolic Syndrome: Clinical Research & Reviews.

[20] Mbunge E, Fashoto S, Akinnuwesi B, Gurajena C, Metfula A, Mashwama P (2020). COVID-19 pandemic in higher education: critical role of emerging technologies in Zimbabwe.

[21] Dobbels F, Van Damme-Lombaert R, Vanhaecke J, De Geest S (2005). Growing pains: non-adherence with the immunosuppressive regimen in adolescent transplant recipients. Pediatr Transplant.

[22] Jimmy B, Jose J (2011). Patient medication adherence: measures in daily practice. Oman medical journal.

[23] Schaecher KL (2013). The importance of treatment adherence in HIV. Am J Manag Care.

[24] Bangsberg DR (2006). Less than 95% adherence to nonnucleoside reverse-transcriptase inhibitor therapy can lead to viral suppression. Clin Infect Dis.

[25] Iacob Simona (2017). “Improving the Adherence to Antiretroviral Therapy, a Difficult but Essential Task for a Successful HIV Treatment—Clinical Points of View and Practical Considerations.”. Frontiers in Pharmacology.

[26] Bangsberg DR (2006). Less than 95% adherence to nonnucleoside reverse-transcriptase inhibitor therapy can lead to viral suppression. Clin Infect Dis.

[27] Paterson DL, Swindells S, Mohr J (2000). Adherence to protease inhibitor therapy and outcomes in patients with HIV infection published correction appears in Ann Intern Med 2002 Feb 5;136(3):253. Ann Intern Med.

[28] Byrd KKB (2019). Antiretroviral Adherence Level Necessary for HIV Viral Suppressionusing Real-World Data. Journal of Acquired Immune Deficiency Syndromes.

[29] O'Halloran Leach E, Lu H, Caballero J, Thomas JE, Spencer EC, Cook RL (2021). Defining the optimal cut-point of self-reported ART adherence to achieve viral suppression in the era of contemporary HIV therapy: a cross-sectional study. AIDS Res Ther.

[30] Pharmacy Quality Alliance (2018). PQA's specialty core measure set.

[31] Crisis 24 online news (2022). Zimbabwe authoroties lift covid -19 related curfew and relax domestic measures.

[32] Government of Zimbabwe Public Health (COVID-19 Prevention, Containment and Treatment) (National Lockdown) (No. 2) (Amendment) Order, 2022 (No. 42).

[33] Government of Zimbabwe Public Health (COVID-19 Prevention, Containment and Treatment) (National Lockdown) (No.2) (Amendment) Order, 2022 (No. 43).

[34] Setia MS (2016). Methodology Series Module 3: Cross-sectional Studies. Indian J Dermatol.

[35] Hudson James I, Pope Harrison G, Glynn Robert J (2005). The Cross-Sectional Cohort Study: An Underutilized Design. Epidemiology.

[36] UNFPA Zimbabwe (2023). What we do: HIV & AIDS.

[37] USAID Center for Development Information and Evaluation (1996). USAID Center for Development Information and Evaluation Issue number 2.

[38] Knobel H, Alonso J, Casado JL (2002). Validation of a simplified medication adherence questionnaire in a large cohort of HIV-infected patients: the GEEMA Study. AIDS.

[39] Ortega Francisco, Plumed J, Valentín MA, Palomo P, Cepeda MA, Aguiar D (2011). Validation on the simplified medication adherence questionnaire (SMAQ) in renal transplant patients on tacrolimus. Nefrología: publicación oficial de la Sociedad Española Nefrologia.

[40] Agala CB, Fried BJ, Thomas JC (2020). Reliability, validity and measurement invariance of the Simplified Medication Adherence Questionnaire (SMAQ) among HIV-positive women in Ethiopia: a quasi-experimental study. BMC Public Health.

[41] Morisky DE, Green LW, Levine DM (1986). Concurrent and predictive validity of a self-reported measure of medication adherence. Med Care.

[42] Bryant F B, Yarnold P R, Grimm L G, Yarnold P R (1996). Principal-components analysis and exploratory and confirmatory factor analysis. Reading and understanding multivariate statistics.

[43] Nunnally J C (1978). Psychometric theory.

[44] Morgan S E, Reichert T, Harrison T R (2017). From numbers to words: Reporting Statistical.

[45] Andrade C (2019). The P Value and Statistical Significance: Misunderstandings, Explanations, Challenges, and Alternatives. Indian J Psychol Med.

[46] Berereton RG (2022). Empirical and statistical p values and Type 1 error rates: Putting it all together. Journal of Chemometrics.

[47] Grabowski B (2016). “P < 0.05” Might Not Mean What You Think: American Statistical Association Clarifies P Values. J Natl Cancer Inst.

[48] Tabachnick BG, Fidell LS (2001). Using Multivariate Statistics.

[49] Health times (2022). Milestone: Zim attains 95-95-95 HIV targets.

[50] Kim J, Lee E, Park BJ (2018). Adherence to antiretroviral therapy and factors affecting low medication adherence among incident HIV-infected individuals during 2009-2016: A nationwide study. Sci Rep.

[51] Hughes CA, Robinson L, Tseng A (2009). New antiretroviral drugs: a review of the efficacy, safety, pharmacokinetics, and resistance profile of tipranavir, darunavir, etravirine, rilpivirine, maraviroc, and raltegravir. Expert Opin Pharmacother.

[52] Birungi C, Haacker M, Taramusi I (2022). Economic implications of COVID-19 for the HIV epidemic and the response in Zimbabwe. Afr J AIDS Res.

[53] Madzima B, Makoni T, Mugurungi O (2022). The impact of the COVID-19 pandemic on people living with HIV in Zimbabwe. Afr J AIDS Res.

[54] Kim Enny Yeon Hee, Barth Shannon K, Monroe Anne K, Ahsan Sarah, Kovacic Janja, Senn Siena, Castel Amanda D (2023). The impact of COVID-19 on the HIV continuum of care: challenges, innovations, and opportunities. Expert Review of Anti-infective Therapy.

[55] Nyashanu M, Chireshe R, Mushawa F, Ekpenyong MS (2021). Exploring the challenges of women taking antiretroviral treatment during the COVID-19 pandemic lockdown in peri-urban Harare, Zimbabwe. Int J Gynaecol Obstet.

[56] Adedigba Segun, Dankyau Musa (2019). Role of Family support in medication adherence in Type 2 Diabetes Mellitus patients at an outpatient setting in Nigeria: A prospective cohort study. International Journal of Medical and Surgical Sciences.

[57] Miller TA, Dimatteo MR (2013). Importance of family/social support and impact on adherence to diabetic therapy. Diabetes Metab Syndr Obes.

[58] Olagbemide OJ, Omosanya O E, Ayodapo A O, Agboola S M, Adeagbo A O, Olukokun T A (2021). Family support and medication adherence among adult type 2 diabetes: Any meeting point?. Annals of African medicine.

[59] Chen X, Du L, Wu R (2020). The effects of family, society and national policy support on treatment adherence among newly diagnosed tuberculosis patients: a cross-sectional study. BMC Infect Dis.

[60] Jankowska-Polańska B, Uchmanowicz I, Dudek K, Mazur G (2016). Relationship between patients' knowledge and medication adherence among patients with hypertension. Patient Prefer Adherence.

[61] Bollini P, Tibaldi G, Testa C, Munizza C (2004). Understanding treatment adherence in affective disorders: a qualitative study. J Psychiatr Ment Health Nurs.

[62] Awwad O, Akour A, Al-Muhaissen S, Morisky D (2015). The influence of patients' knowledge on adherence to their chronic medications: a cross-sectional study in Jordan. Int J Clin Pharm.

[63] Young S, Wheeler AC, McCoy SI, Weiser SD (2014). A review of the role of food insecurity in adherence to care and treatment among adult and pediatric populations living with HIV and AIDS. AIDS Behav.

[64] Zimbabwe National Statistics Agency (2021). Monitoring COVID-19 impacts on households in Zimbabwe Report Number 3, ZIMSTAT 30 June 2021 households in Zimbabwe Report Number 3, ZIMSTAT 30 June 2020.

[65] Imel BE, McClintock HF (2023). Food Security and Medication Adherence in Young and Middle-Aged Adults with Diabetes. Behav Med.

[66] Bautista LE, Vera-Cala LM, Colombo C, Smith P (2012). Symptoms of depression and anxiety and adherence to antihypertensive medication. Am J Hypertens.

[67] DiMatteo MR, Lepper HS, Croghan TW (2000). Depression is a risk factor for noncompliance with medical treatment: meta-analysis of the effects of anxiety and depression on patient adherence. Arch Intern Med.

[68] Goldstein C M, Gathright E C, Garcia S (2017). Relationship between depression and medication adherence in cardiovascular disease: the perfect challenge for the integrated care team. Patient Preference and Adherence.

[69] Panati Dinesh, Chittooru Chandra Sekhar, Madarapu Yethi Raju, Gorantla Ananda Krishna (2023). Effect of depression on treatment adherence among elderly tuberculosis patients: A prospective interventional study, Effect of depression on treatment adherence among elderly tuberculosis patients: A prospective interventional study. Clinical Epidemiology and Global Health.

[70] Grenard J L, Munjas B A, Adams J L, Suttorp M, Maglione M, McGlynn E A, Gellad W F (2011). Depression and medication adherence in the treatment of chronic diseases in the United States: a meta-analysis. Journal of General Internal Medicine.

